# Supplementary material for: Bmp8a is an essential positive regulator of antiviral immunity in zebrafish
Source: Commun Biol. 2021 Mar 9;4:318. doi: 10.1038/s42003-021-01811-0 (PMC7943762; doi:10.1038/s42003-021-01811-0)
Supplement: Supplementary file 1 — Supplementary Information [file 42003_2021_1811_MOESM1_ESM.pdf]

Supplementary Fig.1

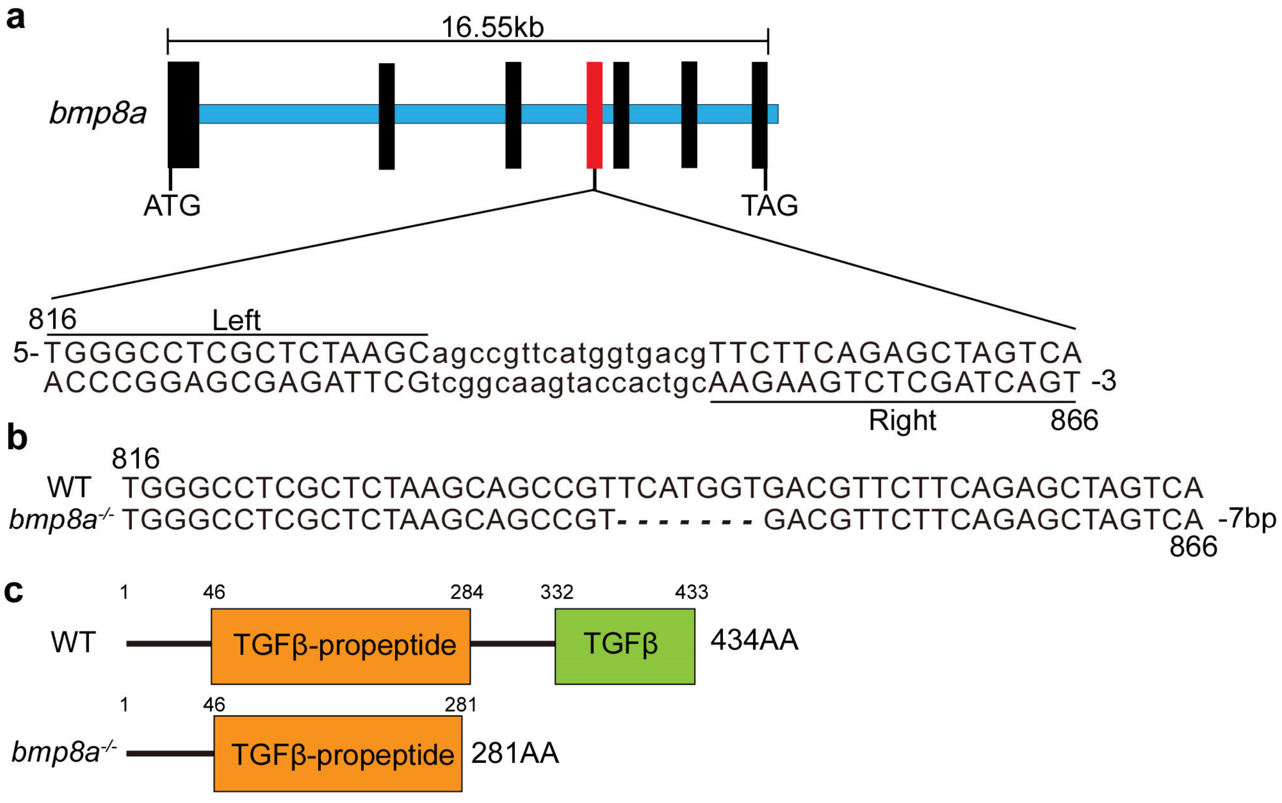

Supplementary Fig. 1 Generation of zebrafish *bmp8a* mutant alleles using TALENs technology. **a** Partial structure and sequence of the *bmp8a* gene, showing the binding sites (underlined) of the left and right TALENs. **b** Sequences of *bmp8a* homozygous mutant. Deletions of 7 bp are indicated by dashes letters. The sequences of *bmp8a* in wild type (WT) zebrafish were used as a control. **c** Bmp8a in WT, 434 amino acids (aa) long, contains a TGFβ-propeptide domain (yellow) and a TGFβ domain (green). The *bmp8a*$^{-/-}$ encodes a truncated 281-amino-acid-long polypeptide that contains a partial TGFβ-propeptide domains (yellow) followed by a frameshift leading to a premature stop codon.

Supplementary Fig. 2

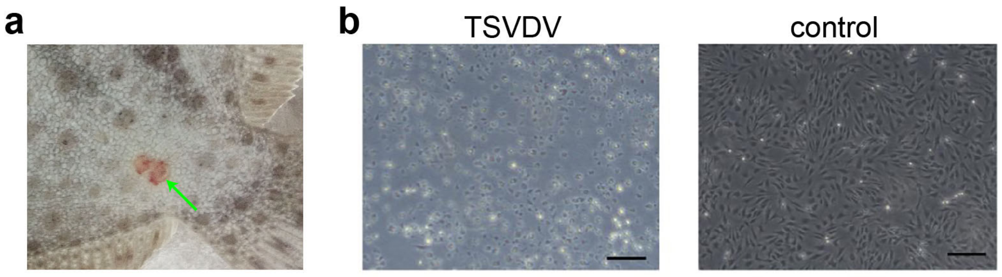

Supplementary Fig. 2 The virus (TSVDV) was isolated from the focal site of turbot skin. a Skin ulceration appeared on the skin surface (green arrow) of turbot infected with TSVDV[1]. **b** The FG cells had obvious cytopathic effect (CPE) after TSVDV infection.

Supplementary Fig. 3

```
-1439 TCACTTTCGTCAATTGGTGAAGTTTGTTCCTCATGACTGGCTTGCTTGGTTTGGGACTTGTGGAGCT
-1372 GCGCATCGATGGATTTGTTCTTCAGTGTTTGGACTTTCAGTAGGGAAAATTAAACC
                                                        GAS motif
-1316 ACACTGAACTGAACTTCAACTCTGAAAACTGGTCTGACTCCGTTTCAATTTACTAGAACTT
                                                            P2
-1255 CTCTGTTAAGCTGATCTGACACAATCTACATTGTAAAAGTGCTATAGAAATAAAGATGAAATAAATT
-1188 TTTATAATATTTACCTAAGTACACTGCTCCAAAAAATAAAGGGAACACTTAAACAACACAATATAA
-1122 CCATGTGTTGCCTTTATCTTTTTAGCAGTATGTTTATGCATTTGGTGCTGTAATGAACAACTACTAAT
-1054 ATAACAATCTGAATATTCCTAACAGCCACTCTAAAAGTTTAAATAATATATTTTCAAGTGCACACGA
 -987 TATCAGTATTGTCACTATCACAATACATTGAATTGTTGAGTATCAACATACGTCTCATACACAGTGA
 -920 TCTCTCACTGCTTGGCTACCGCTGAACCCACAGAATACAAATTTAGTTTTCATATTACTGTAGCCAC
 -853 ACAAACTCCGTCCCATTCTGTGCAAAGACTGGATCACATCTCCACTCCAAACCACATGATAATTGT
 -787 CAGCAATTTGTGAGGTATGTGTAGAAGATGAGACGGTTTGATAGGGCAGATGGAGACTGAGGTGA
 -722 GAAGGGTTCAGGATAGAGAAAAGGGCGGAGGTTTGAGATGATGGAAACCCTCCCTCTGCGTAAG
 -658 CCTGTTTCTTGGAGGGAAGGAGCTCGCCTGTGTTCAGAGTAACCCTCCAGCGCAAGCAGCACACA
 -593 AGCTAAGAATACCACAGCTCAACAAAGTTCAGCGGTGAACCAATACAATTCCTTCTCAGAGTTTTC
 -527 AGGAGCCTTGGATTTTCCATCCTTTGGATTATTTGTTTTTTTTTTTAAAGCTCAACTTTTCATGGATTA
 -458 CCTCAAGACAGTTTTGTTTTTCAATTAAACAGAGGATTTCACCAGTGAAGAGTTCTCAAAAGAAGC
 -392 AGGTGTGCTTTTTAAAGGTTGACCGCTGTTTTTAGTTACTCTACCCAAAGACTTTCCATTCAACATT
 -325 GTGCTTTGTCCTTCCAACATCCAAAATCCAGTTTCCAGGATATATTGTCATCCATCTTTGTAAGTGC
 -258 ACTATAAATTATGAGATTGCTTTGGTAAATAAAGTGACGGCATGAGGAACGACTGTCAAGAGGCG
 -193 ATAAACTGAGCAAGTAAACATTGCACTCTTCCAAATAACGATGACTGCTGTGGAATCTGAGTGGCT
                                                          GAS motif
 -127 TAGACGTTCGAGTGTTGTTCGGGGTTGAACTTCGAGACGGTAGTCAGACCATTCCGGGAAAACCA
                                                              P1
  -62 ACGTCGGGTGAACAGCTAAAAGGACAAATATCTTTACAGACGGCTGGGCTCTTCGCTACACCA
      +1
    1 ATGGACAGACACGAGGTT
```

Supplementary Fig. 3 Sequence analysis of *bmp8a* promoter region. Two GAS motifs (P1 and P2) have been found in the *bmp8a* promoter region, which were underlined. The transcriptional start site was designated by +1.

Supplementary Fig. 4

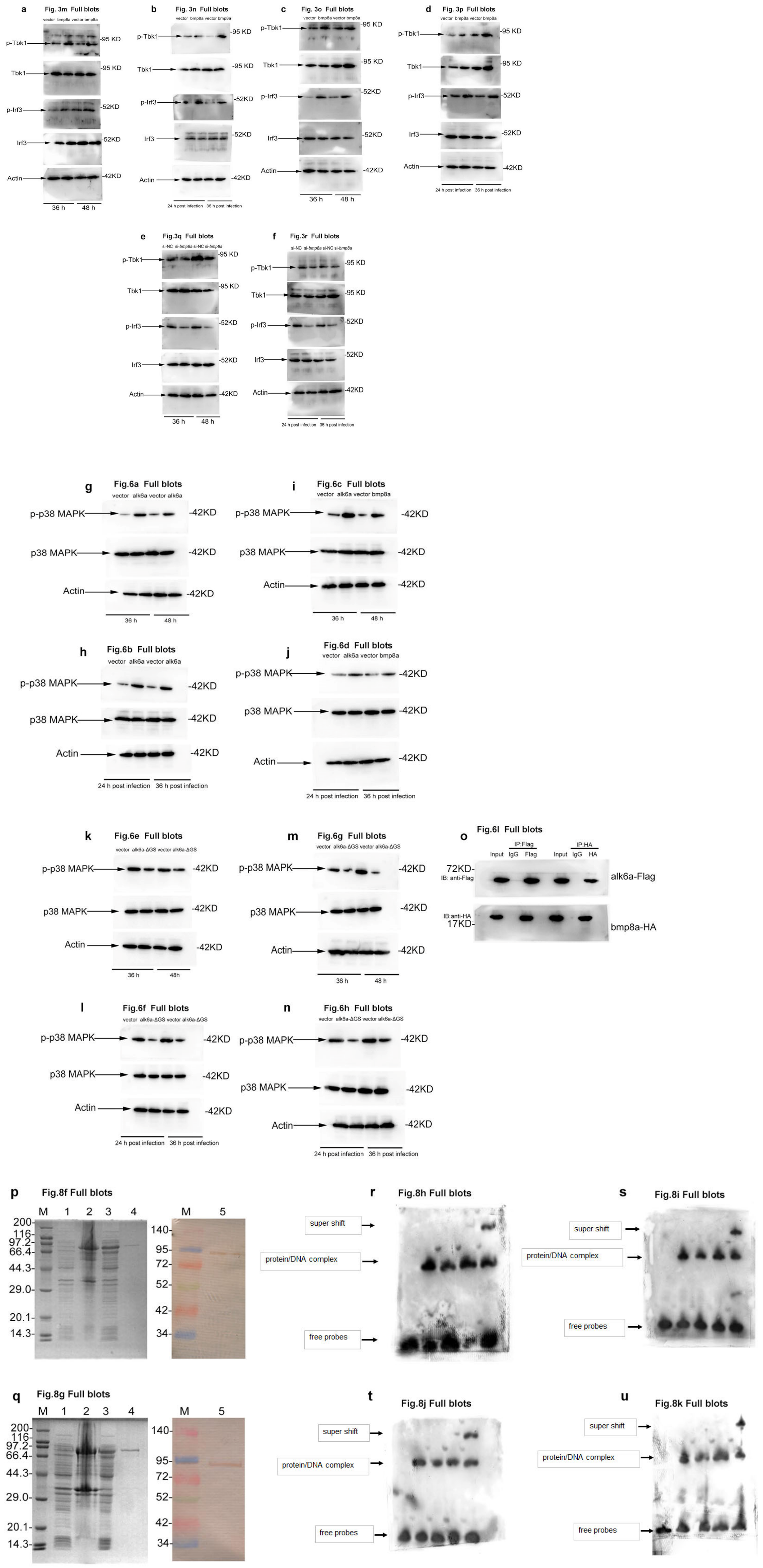

Supplementary Fig. 4 The uncropped blots in this study. a Complete western blots illustrated in Fig. 3m. **b** Complete western blots illustrated in Fig. 3n. **c** Complete western blots illustrated in Fig. 3o. **d** Complete western blots illustrated in Fig. 3p. **e** Complete western blots illustrated in Fig. 3q. **f** Complete western blots illustrated in Fig. 3r. **g** Complete western blots illustrated in Fig. 6a. **h** Complete western blots illustrated in Fig. 6b. **i** Complete western blots illustrated in Fig. 6c. **j** Complete western blots illustrated in Fig. 6d. **k** Complete western blots illustrated in Fig. 6e. **l** Complete western blots illustrated in Fig. 6f. **m** Complete western blots illustrated in Fig. 6g. **n** Complete western blots illustrated in Fig. 6h. **o** Complete western blots illustrated in Fig. 6l. **p** Complete blots illustrated in Fig. 8f. **q** Complete blots illustrated in Fig. 8g. **r** Complete blots illustrated in Fig. 8h. **s** Complete blots illustrated in Fig. 8i. **t** Complete blots illustrated in Fig. 8j. **u** Complete blots illustrated in Fig. 8k.

Supplementary Table 1

Primer names	Sequences (5' to 3')	Applications
irf3-F	GCTGTTCGTGCCCTGGTTGTATG	qRT-PCR for zebrafish
irf3-R	GCACATCGTCGCTGTTGGAGTC	
irf7-F	GCTGGAATTGCTCAACTTGGTGG	
irf7-R	CAGTCTGTGGTCGGGAAGCGTAT	
mx-F	CCTGGCAGAATCTATGCTGAAAAC	
mx-R	AAGGCAGTTTTATTTTCGGTTGTG	
ifnφ1-F	GTGGAGGACCAGGTGAAGTT	
ifnφ1-R	GATTGACCCTTGCGTTGC	
ifnφ3-F	TCTATACCATTCCCGCAAAA	
ifnφ3-R	GGATCACCGAAGTTCTCAAAC	
tbk1-F	TGATTGGAGACGACGGATTC	
tbk1-R	CCACTCGATCTTCCCATTTTC	
alk2-F	TGGGTTGGTGCTGTGGGAGA	
alk2-R	CGGGTGAGCGGTCAGTAGTT	
alk3-F	GCATCACGCTTAACCATCC	
alk3-R	TCCCAAATCCACCAGAACA	
alk6a-F	AGTGTCTCCGACAGATGGGG	
alk6a-R	TGTGAGTTCAGGGTTTGGGT	
bmpr2a-F	GAGATACCAAAACTCGACCCG	
bmpr2a-R	TCCTTGTGACCCCGAAACC	
bmpr2b-F	GTGATGATGGGCGAGTGTAA	
bmpr2b-R	CGATAAAGCGAGCAATGTTT	
actr2a-F	TTGATGGCGGGTTCTATGC	
actr2a-R	TCCTCGGATTCAGCGAGTG	
actr2b-F	TGCTATTTTACCACGACCCT	
actr2b-R	CACAACACCAGATAAACCTTGA	
bmp8a-F	TCGCTGGCTTCTCCATCCT	
bmp8a-R	ATCACATTCCCCGTCACAA	
actb1-F	CCGTGACATCAAGGAGAAGC	
actb1-R	TACCGCAAGATTCCATACCC	
EPC *actin*-F	TGTTCCAGCCATCCTTCTTG	qRT-PCR for EPC cells
EPC *actin*-R	TGATTTTCATTGTGCTGGGG	
EPC *ifn*-F	ATGAAAACTCAAATGTGGACGTA	
EPC *ifn*-R	GATAGTTTCCACCCATTTCCTTAA	
EPC *irf3*-F	AACAAGAATGACACTGCGGA	
EPC *irf3*-R	AACTCGGGAGGGACTTTCAT	
EPC *irf7*-F	AAAGTCTTCGTCAGCACCAGCG	
EPC *irf7*-R	CTCTCCGAAGCACAGGTAGATGGT	
EPC *tbk1*-F	TCAGAAGTTTGAGAACGGGAAGA	
EPC *tbk1*-R	CGTAGACCACGATGCGGTGTAAG	
EPC *mx*-F	GGCTGGAGCAGGTGTTGGTATC	
EPC *mx*-R	TCCACCAGGTCCGGCTTTGTTAA	
GCRV VP5-F	CTCCCCGTGAGCGTATTT	qRT-PCR for GCRV
GCRV VP5-R	GTTAGCAGCGGTAGTGACTTG	
P1 biotin probe-F	biotin-AGACCATTCCGGGAAAACCAA	EMSA
P1 biotin probe-R	biotin-TTGGTTTTCCCGGAATGGTCT	
P1 non-biotin probe-F	AGACCATTCCGGGAAAACCAA	
P1 non-biotin probe-R	TTGGTTTTCCCGGAATGGTCT	
P1 mutant non-biotin probe-F	AGACCAGCCCGGGGCAACCAA	
P1 mutant non-biotin probe-R	TTGGTTGCCCCGGGCTGGTCT	
P2 biotin probe-F	biotin-TTCAATTTACTAGAACTTCTC	
P2 biotin probe-R	biotin-GAGAAGTTCTAGTAAATTGAA	
P2 non-biotin probe-F	TTCAATTTACTAGAACTTCTC	
P2 non-biotin probe-R	GAGAAGTTCTAGTAAATTGAA	
P2 mutant non-biotin probe-F	TTCAATGCACTAGGCCTTCTC	
P2 mutant non-biotin probe-R	GAGAAGGCCTAGTGCATTGAA	
bmp8a talen-F	TGGGCCTCGCTCTAAGC	TALEN
bmp8a talen-R	ACTGATCGAGACTTCTT	
bmp8a F1-F	GGAACTCTGAATCTGCGTCT	
bmp8a R1-R	TCACAGGAGGGCGAATAG	
bmp8a F2-F	GACAGACATTCAGGCACGTA	
bmp8a R2-R	GCCGTCCACTGCTATGAT	

Supplementary Table 1. Oligonucleotides used in this study.

Supplementary Table 2

Primer names	Sequences (5' to 3')	Applications
pcDNA3.1-bmp8a-F	CGGGATCCGCCACCATGGACAGACACGAGGT	Eukaryotic expression
pcDNA3.1-bmp8a-R	GGAATTCCTATAAACAGCCACAATTC	
pcDNA3.1-stat1a-F	GGGGTACCGCCACCATGACTCAGTGGTTGGAGC	
pcDNA3.1-stat1a-R	GCTCTAGACTATGTCATTGTCATCAAG	
pcDNA3.1-stat1b-F	GGGGTACCGCCACCATGACGCTCTGGAACCA	
pcDNA3.1-stat1b-R	GCTCTAGACTAAAACCTTGCAACGGGT	
pcDNA3.1-alk2-F	CCCAAGCTTGCCACCATGGGGCATTGCAGCAC	
pcDNA3.1-alk2-R	CGGGATCCTCAGCAGTCGGTTTTGCCCTT	
pcDNA3.1-alk3-F	CCCAAGCTTGCCACCATGCGTCAGCTTTTGT	
pcDNA3.1-alk3-R	CGGGATCCTCAGATTTTAATGTCTTGAGAT	
pcDNA3.1-alk6a-F	CGGGATCCGCCACCATGGATCTCTATAGGA	
pcDNA3.1-alk6a-R	GCTCTAGATCAGACTTTAATGTCCTGGGAT	
pcDNA3.1-actr2a-F	CCCAAGCTTGCCACCATGGGACCTGCAACAAA	
pcDNA3.1-actr2a-R	GGAATTCTCATAGACTAGACTCCTTTGGG	
pcDNA3.1-actr2b-F	CCCAAGCTTGCCACCATGTTCGCTTCTCTGCTC	
pcDNA3.1-actr2b-R	GGAATTCTCAGATGCTGGACTCTTTGGGC	
pcDNA3.1-bmpr2a-F	GGAATTCGCCACCATGGCAGTTGAAGGCCGAATA	
pcDNA3.1-bmpr2a-R	GCTCTAGATCAAAAGTCACAGTCATTAGGATCTG	
pcDNA3.1-bmpr2b-F	GGAATTCGCCACCATGAGAGTGCGGATCCTAAAC	
pcDNA3.1-bmpr2b-R	GCTCTAGATCACAGGCAGGTCATATCTGG	
pET-28a-stat1a-F	GGAATTCATGACTCAGTGGTTGGAGCTTC	Prokaryotic expression
pET-28a-stat1a-R	CCCAAGCTTTGTCATTGTCATCAAGTCTGC	
pET-28a-stat1b-F	CGGGATCCATGACGCTCTGGAACCAGCTG	
pET-28a-stat1b-R	CCGCTCGAGAAACCTTGCAACGGGTCTTG	
pGL3-bmp8a-promoter-F	GGGGTACCTCACTTTCGTCAATTGGTGAA	Luciferase assay
pGL3-bmp8a-promoter-R	CCGCTCGAGTGGTGTAGCGAAGAGCCCAGC	
pGL3-bmp8a-promoter-ΔP1-F	GGTCTGACTCCGTTTCAATTCTCTGTTAAGCTGATCTGACACAA	
pGL3-bmp8a-promoter-ΔP1-R	TTGAAACGGAGTCAGACCAGTTTTCAGAGTTGA	
pGL3-bmp8a-promoter-ΔP2-F	TAGTCAGACCACCAACGTCGGGTGAACAGCTAA	
pGL3-bmp8a-promoter-ΔP2-R	ACGTTGGTGGTCTGACTACCGTCTCGAAGTTCA	
pGL3-IFNφ1-promoter-F	TCTCGAGAAGTGAAGTGAAAGTGCA	
pGL3-IFNφ1-promoter-R	CAAGCTTGTTCTCATCTTTGCGTGT	
pGL3-IFNφ3-promoter-F	GGGTACCGTTCACTGAGCCTGCATG	
pGL3-IFNφ3-promoter-R	TCTCGAGGATGCCTATACGGAATCA	
pGL3-EPC IFN-promoter-F	GGGGTACCGACCTTGAAATACTTTGGAATCAGGT	
pGL3-EPC IFN-promoter-R	CCCAAGCTTGCACAAACATATACGTCCACATT	
pCMV-bmp8a-HA-F	AGCCCGGGCGGATCCAAGCTTGCCACCATGGACAGACACGA	CO-IP
pCMV-bmp8a-HA-R	ATCGAATTCCTGCAGAAGCTTTAAACAGCCACAATTCTTGACCA	
pCMV-alk6a-Flag-F	CGCTCTAGCCCGGGCGGATCCGCCACCATGGATCTCTATAGGAGC	
pCMV-alk6a-Flag-R	TTCCTGCAGAAGCTTGGATCCGACTTTAATGTCCTGGGATTCAGAC	
pcDNA3.1-alk6a-ΔGS-F	ATAGAGCAATCCCAGCTCCCTCTACTGGTGCAGCG	Dominant negative mutant
pcDNA3.1-alk6a-ΔGS-R	GAGCTGGGATTGCTCTATGAGATCCCGCAGAGA	
pcDNA3.1-irf3DN-F	CTTGGTACCGAGCTCGGATCCGCCACCATGATCATTTTGAATCC	
pcDNA3.1-irf3DN-R	CCACACTGGACTAGTGGATCCTTAGCAGAGCTCCATCATTTGCT	
pcDNA3.1-irf7DN-F	CTTGGTACCGAGCTCGGATCCGCCACCATGAAGAGTGCGAT	
pcDNA3.1-irf7DN-R	CCACACTGGACTAGTGGATCCTTATTCCACTGAAGGCAGACCC	
pcDNA3.1-stat1a-ΔC-F	CTTGGTACCGAGCTCGGATCCGCCACCATGACTCAGTGGTTG	
pcDNA3.1-stat1a-ΔC-R	CCACACTGGACTAGTGGATCCTCAGTCGGCGGCCATTACG	
pcDNA3.1-stat1b-ΔC-F	CTTGGTACCGAGCTCGGATCCGCCACCATGACGCTCTGGA	
pcDNA3.1-stat1b-ΔC-R	CCACACTGGACTAGTGGATCCTCAATCTTTTGGGATGTTCGG	
pcDNA3.1-tbk1 K38M-F	CCGTCATGGTGTTCAACAACCTGGTGTTCAACAACCTGAGTTTCCTG	
pcDNA3.1-tbk1 K38M-R	TGTTGAACACCATGACGGCGTACAGATTGACGGCGTACAGATCGCC	

Supplementary Table 2. Oligonucleotides used in this study.

Supplementary References

1. Qin, L., Wang, Y. G. & Zhang, Z. Multiple verrucous protrusions on skin in turbot Scophthalmus maximus. *J. Dalian Ocean Univ.* **23**, 479–483 (2008).
