# Supplementary material for: Bmp8a is an essential positive regulator of antiviral immunity in zebrafish
Source: Commun Biol. 2021 Mar 9;4:318. doi: 10.1038/s42003-021-01811-0 (PMC7943762; doi:10.1038/s42003-021-01811-0)
Supplement: Supplementary file 3 — Description of Additional Supplementary Files [file 42003_2021_1811_MOESM3_ESM.pdf]

Description of Additional Supplementary Files

File Name: Supplementary Data 1

Description: Source data underlying plots shown in the figures
