# Supplementary material for: Bmp8a is an essential positive regulator of antiviral immunity in zebrafish
Source: Commun Biol. 2021 Mar 9;4:318. doi: 10.1038/s42003-021-01811-0 (PMC7943762; doi:10.1038/s42003-021-01811-0)
Supplement: Supplementary file 5 — Reporting Summary [file 42003_2021_1811_MOESM5_ESM.pdf]

nature research

Corresponding author(s): zhenhui liu

Last updated by author(s): Dec 13, 2020

Reporting Summary

Nature Research wishes to improve the reproducibility of the work that we publish. This form provides structure for consistency and transparency in reporting. For further information on Nature Research policies, see our Editorial Policies and the Editorial Policy Checklist.

Statistics

For all statistical analyses, confirm that the following items are present in the figure legend, table legend, main text, or Methods section.

n/a	Confirmed	
☐	☒	The exact sample size (*n*) for each experimental group/condition, given as a discrete number and unit of measurement
☐	☒	A statement on whether measurements were taken from distinct samples or whether the same sample was measured repeatedly
☐	☒	The statistical test(s) used AND whether they are one- or two-sided<br>*Only common tests should be described solely by name; describe more complex techniques in the Methods section.*
☒	☐	A description of all covariates tested
☒	☐	A description of any assumptions or corrections, such as tests of normality and adjustment for multiple comparisons
☒	☐	A full description of the statistical parameters including central tendency (e.g. means) or other basic estimates (e.g. regression coefficient) AND variation (e.g. standard deviation) or associated estimates of uncertainty (e.g. confidence intervals)
☐	☒	For null hypothesis testing, the test statistic (e.g. *F*, *t*, *r*) with confidence intervals, effect sizes, degrees of freedom and *P* value noted<br>*Give P values as exact values whenever suitable.*
☒	☐	For Bayesian analysis, information on the choice of priors and Markov chain Monte Carlo settings
☒	☐	For hierarchical and complex designs, identification of the appropriate level for tests and full reporting of outcomes
☒	☐	Estimates of effect sizes (e.g. Cohen's *d*, Pearson's *r*), indicating how they were calculated

Our web collection on statistics for biologists contains articles on many of the points above.

Software and code

Policy information about availability of computer code

Data collection	n/a
Data analysis	GraphPad Prism 8.0.1

For manuscripts utilizing custom algorithms or software that are central to the research but not yet described in published literature, software must be made available to editors and reviewers. We strongly encourage code deposition in a community repository (e.g. GitHub). See the Nature Research guidelines for submitting code & software for further information.

Data

Policy information about availability of data

All manuscripts must include a data availability statement. This statement should provide the following information, where applicable:

- Accession codes, unique identifiers, or web links for publicly available datasets
- A list of figures that have associated raw data
- A description of any restrictions on data availability

All relevant data are available from the authors upon request and the corresponding author will be responsible for replying to the request.

Field-specific reporting

Life sciences study design

All studies must disclose on these points even when the disclosure is negative.

Sample size	n/a
Data exclusions	No Data was excluded
Replication	Representative experiments have been repeated at least two to three times and all replicates were successful.
Randomization	n/a
Blinding	n/a

Reporting for specific materials, systems and methods

We require information from authors about some types of materials, experimental systems and methods used in many studies. Here, indicate whether each material, system or method listed is relevant to your study. If you are not sure if a list item applies to your research, read the appropriate section before selecting a response.

Materials & experimental systems

n/a	Involved in the study	
☐	☒	Antibodies
☐	☒	Eukaryotic cell lines
☒	☐	Palaeontology and archaeology
☐	☒	Animals and other organisms
☒	☐	Human research participants
☒	☐	Clinical data
☒	☐	Dual use research of concern

Methods

n/a	Involved in the study	
☒	☐	ChIP-seq
☒	☐	Flow cytometry
☒	☐	MRI-based neuroimaging

Antibodies

Antibodies used	TBK1; p-TBK1(Ser172); IRF3; p-IRF3 (Ser386); p38MAPK; p-p38MAPK (Thr180 + Tyr182); Actin; HA tag; Flag tag; His tag; goat anti-rabbit IgG HRP secondary antibody; VeriBlot for IP Detection
Validation	anti-TBK1 (1:1000, CST, 3504T)<br>anti-p-TBK1(Ser172) (1:1000, CST, 5483T)<br>anti-IRF3 (1:1000, Bioss, bs-2993R)<br>anti-p-IRF3 (Ser386) (1:1000, Bioss, bsm-52170R)<br>anti-p38MAPK (1:1000, Bioss, bs-0637R)<br>anti-p-p38MAPK (Thr180 + Tyr182) (1:1000, Bioss, bs-2210R),<br>anti-Actin (1:2000, Bioss, bs-0061R)<br>anti-HA tag (1:1000, Beyotime, AH158)<br>anti-Flag tag (1:1000, Beyotime, AF519)<br>anti-His tag (1:5000, CWBIO, CW0285)<br>anti-goat anti-rabbit IgG HRP secondary antibody (1:8000, CWBIO, CW0103S)<br>anti-VeriBlot for IP Detection (1:5000, Abcam, ab131366)

Eukaryotic cell lines

Policy information about cell lines

Cell line source(s)	ZFL and EPC cells were purchased form CZRC. FG cells were generated in the our lab.
Authentication	The cell lines were authenticated.
Mycoplasma contamination	All cell lines tested negative for mycoplasma contaminatiom.
Commonly misidentified lines (See ICLAC register)	No misidentified cell lines used in the study.

Animals and other organisms

Policy information about studies involving animals; ARRIVE guidelines recommended for reporting animal research

Laboratory animals	Zebrafish, male, 4-6 months.
Wild animals	The study did not involve wild animals.
Field-collected samples	this Study did not involve sample collected from the field.
Ethics oversight	The animals used in the experiment followed the ethical guidelines established by the Institutional Animal Care and Use Committee of the Ocean University of China (permit number, SD2007695).

Note that full information on the approval of the study protocol must also be provided in the manuscript.
